# Thoracic Outlet Syndrome: Single Center Experience on Robotic Assisted First Rib Resection and Literature Review

**DOI:** 10.3389/fsurg.2022.848972

**Published:** 2022-03-08

**Authors:** Andreas Gkikas, Savvas Lampridis, Davide Patrini, Peter B. Kestenholz, Luis Filipe Azenha, Gregor Jan Kocher, Marco Scarci, Fabrizio Minervini

**Affiliations:** ^1^Department of Cardiothoracic Surgery, Royal Victoria Hospital, Belfast, United Kingdom; ^2^Department of Thoracic Surgery, 424 General Military Hospital, Thessaloniki, Greece; ^3^Department of Thoracic Surgery, University College London Hospitals, London, United Kingdom; ^4^Department of Thoracic Surgery, Cantonal Hospital Lucerne, Lucerne, Switzerland; ^5^Division of Thoracic Surgery, Bern University Hospital, University of Bern, Bern, Switzerland; ^6^Department of Thoracic Surgery, Imperial College Healthcare NHS Trust, London, United Kingdom

**Keywords:** thoracic outlet syndrome, first rib resection, robotic assisted resection, thoracic outlet, robotic thoracic surgery (RATS)

## Abstract

**Background:**

Thoracic outlet syndrome (TOS) is a pathological condition caused by a narrowing between the clavicle and first rib leading to a compression of the neurovascular bundle to the upper extremity. The incidence of TOS is probably nowadays underestimated because the diagnosis could be very challenging without a thorough clinical examination along with appropriate clinical testing. Beside traditional supra-, infraclavicular or transaxillary approaches, the robotic assisted first rib resection has been gaining importance in the last few years.

**Methods:**

We conducted a retrospective cohort analysis of all patients who underwent robotic assisted first rib resection due to TOS at Lucerne Cantonal Hospital and then we performed a narrative review of the English literature using PubMed, Cochrane Database of Systematic Reviews and Scopus.

**Results:**

Between June 2020 and November 2021, eleven robotic assisted first rib resections were performed due to TOS at Lucerne Cantonal Hospital. Median length of stay was 2 days (Standard Deviation: +/– 0.67 days). Median surgery time was 180 min (Standard Deviation: +/– 36.5). No intra-operative complications were reported.

**Conclusions:**

Robotic assisted first rib resection could represent a safe and feasible option in expert hands for the treatment of thoracic outlet syndrome.

## Introduction

Thoracic Outlet Syndrome (TOS) includes a variety of disorders that cause compression of neurovascular structures as they travel from the posterior triangle of the neck to the axilla (1). This can result in subclinical manifestations to even debilitating symptoms in the upper extremity. The anatomical borders of thoracic outlet are the sternum medially, the clavicle anteriorly, the insertion of the pectoralis minor muscle onto the coracoid process of the humerus laterally and the first thoracic rib posteriorly (1).

The prevalence of TOS in the general population should be interpreted with caution due to lack of epidemiological data in the literature (1, 2). It is commonly diagnosed in young adults aged 20–50 years and affects women 3–4 times more frequently than men (3–6). The most frequently quoted estimate of its incidence ranges from three to 80 per 1,000 people (3–6). However, evidence suggests that this estimate, is probably replicated in the literature without updated or valid epidemiological studies (7, 8). In a more recent retrospective analysis of an established prospective database from the United States, the authors support that TOS is actually far rarer with an incidence of 2–4/100,000 people per year (9).

Apart from its prevalence, there are many other aspects of the syndrome which have been disputed in medical literature. Those include its etiology and classification, the symptoms which are typically present, the diagnostic criteria (both clinical and investigations) and finally its management and treatment options (1, 4, 5). Surgical treatment is mostly restricted for patients with persisting and severe symptoms following failed conservative management (4, 5). The main goal of the surgical interventions for TOS is to decompress the thoracic outlet in order to avoid excess pressure upon the structures of the neurovascular bundle. To achieve this, resection of the first rib is considered the most frequent operation (4, 5). The most traditional operative approaches in TOS are supraclavicular and transaxillary (4, 5, 10–12). However, minimally invasive approaches have been developed over the last decades with Robotic assisted thoracoscopic surgery (RATS) constantly gaining supporters in the last years (12).

## Methods

We conducted a retrospective cohort analysis of all patients who underwent robotic first rib resection due to TOS. We analyzed clinical and patient outcomes. The retrospective analysis was approved by the institutional review board and individual consent was waived.

In the discussion session we conducted a narrative literature review using PubMed, Cochrane Database of Systematic Reviews and Scopus. Our search strategy was comprised of the following key words and Boolean operators: (“Thoracic Outlet Syndrome” OR “TOS” OR “First rib resection”) AND (Robot or RATS).

We reviewed all relevant papers and linked articles published between 1964 and 2021. Due to the narrative design of the review a certain subjectivity in choice of studies included is likely.

### Statistical Analysis

Continuous variables are reported as mean in normally distributed data; discrete variables are reported as numbers. All data were de-identified with a sequentially-generated study identification code, encrypted, and stored to the central Lucerne site for analysis. SPSS 25.0 (www.ibm.com) was used for statistical analysis.

## Results

**Figure 1:**
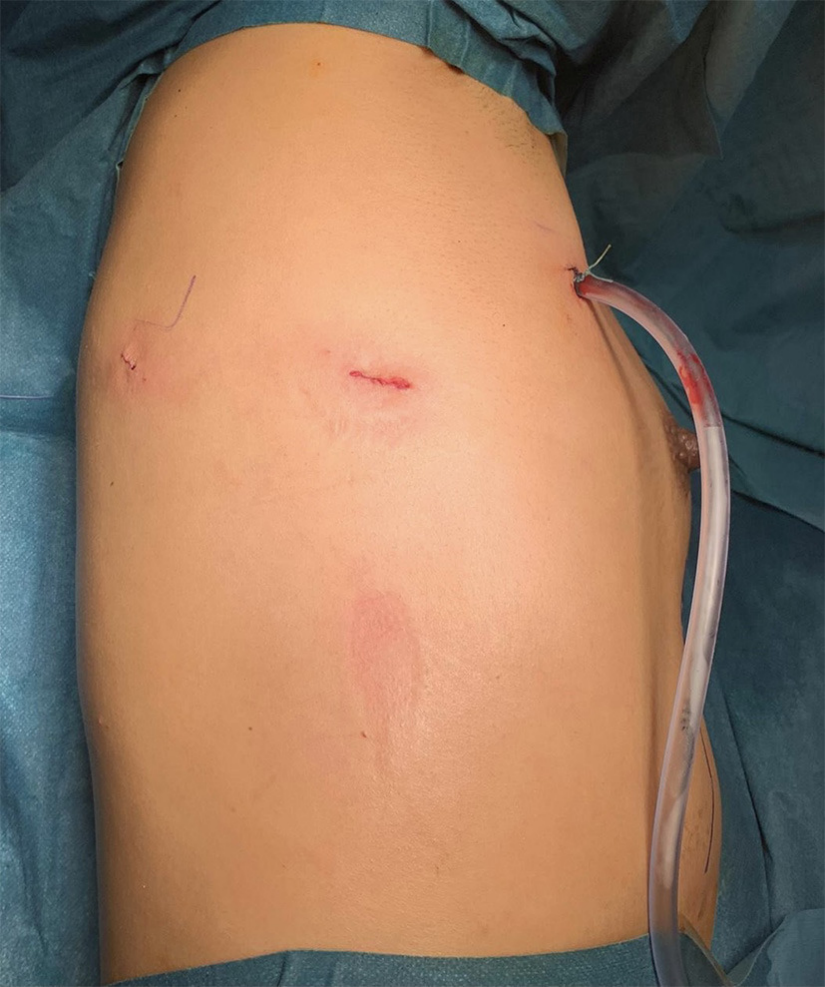
Triportal approach for robotic 1st rib resection.

**Figure 2:**
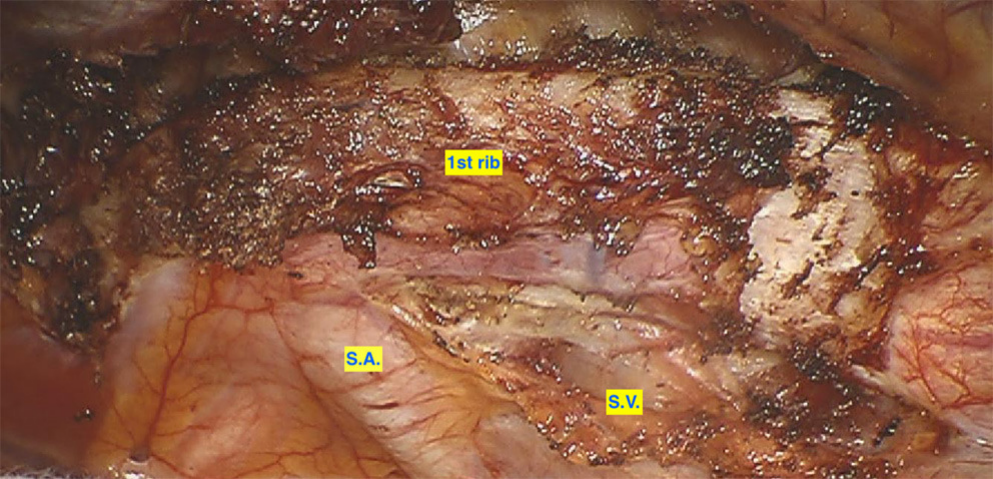
Preparation of the first rib before resection. SA, Subclavian Artery; SV, Subclavian Vein.

**Figure 3:**
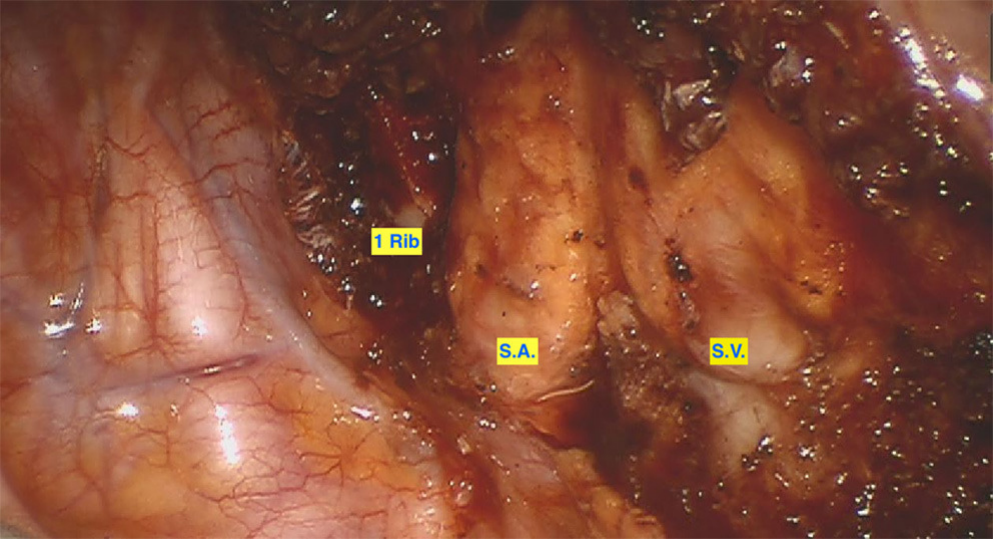
Visualization of the subclavian artery and vein after 1st rib resection. 1 Rib: dorsal margin of the resected 1st rib. SA, Subclavian Artery; SV, Subclavian Vein.

The single center experience of the thoracic surgery department of the Cantonal Hospital of Lucerne, Switzerland, suggests that a robotic minimally invasive triportal approach (Figures 1–3) is feasible and safe. Between June 2020 and November 2021, eleven robotic assisted first rib resections were performed due to TOS (2 arterial TOS, 8 venous TOS and 1 neurogenic TOS; 8 were on the right side, 3 on left side). Median age of the patients was 28 years (Standard Deviation: 9.55), median length of stay was 2 days (Standard Deviation: +/– 0.67 days). Median surgery time was 180 min (Standard Deviation: +/– 36.5). No intra-operative complications were reported. We observed only 1 post-operative grade IIIa complication according to the Clavien-Dindo Classification (pneumothorax requiring chest tube) (13). At 2 weeks post-operative follow up all the patients had a total resolution of symptoms with a mean self-reported Pain visual analog scale (VAS) score of 0.6 (Standard Deviation: +/– 1.99).

The mean score for the EQ-5D-5L collected at that time was 0.95 (Standard Deviation: +/– 0.12).

## Discussion

### Categories and Etiology

As it has been already described in the Introduction, TOS results from compression of the neurovascular bundle (1, 4, 5). This is comprised by the brachial plexus and the subclavian and axillary vessels (1). It courses through three narrowed spaces within the thoracic outlet. Each of these compartments is a potential site of neurovascular compression. Starting from the medial site, the first space is the scalene triangle. This is defined as the triangular compartment formed by the upper border of the first rib, the anterior and middle scalene muscles. As the bundle travels anterior-laterally it goes through the costoclavicular space which is bordered by the first rib, the clavicle and the scapula. Just before it enters the axilla, the bundle runs beneath the coracoid process, deep to the pectoralis minor tendon, through the subcoracoid space. These three spaces become even more constricted when the upper limb is abducted or externally rotated at the shoulder joint.

A significant problem in TOS research is the inconsistency in diagnosis and classification of the syndrome in the literature (4, 5). TOS has been categorized according to multiple different parameters. These include the predominant symptoms, which space in the thoracic outlet is narrowed or the etiology of bundle compression (soft-tissue or osseous abnormalities) (2, 4, 5). However, the most widely used classification of TOS is into 3 main categories based on which component of the neurovascular bundle is being compressed. These are, Arterial TOS (A-TOS), Venous TOS (V-TOS) and Neurogenic TOS (N-TOS). The latter class is frequently subdivided to true NTOS and disputed NTOS according to the presence or absence of objective findings related with nerve compression (4, 5). Some researchers also report that traumatic neurovascular TOS comprises a fourth clinical entity of the TOS spectrum. This category includes conditions in which neurological and vascular features coexist following clavicular trauma. On the other hand, other authors argue that TOS should be classified in 5 categories by recognizing true NTOS and disputed NTOS as entirely separate classifications (4).

N-TOS has historically been regarded as the most frequently diagnosed category of TOS. Even though, most publications report that it accounts for 90–95% of TOS cases, there have been several publications over the last decade which place this estimate at around 70–80% (8, 9, 11, 14, 15). The True NTOS has been reported in the literature under different terminology such as “classic TOS” and “cervical rib and band syndrome” (3, 4, 16). Its predominant etiology is a fibrous band that extends from the first thoracic rib to a bony anomaly of the last cervical vertebra which is either a cervical rib or an elongated transverse process. This results in compression of the lower supraclavicular brachial plexus which elicits symptoms (16). Its prevalence is estimated at around 1 per 1,000,000 people and it represents only 1% of the NTOS cases (17).

On the other hand, disputed NTOS appears to be much more common and accounts for 95–99% of NTOS (18, 19). There have been reports in the literature of significantly high incidence in the general population that reach even 8% (20, 21). However, this finding has been questioned from the results of a retrospective analysis from a recent prospective database and the authors report that it is estimated at around 2 and 3 per 100,000 people per year (9). Even though disputed NTOS is recognized as a clinical entity, the actual details on its pathogenesis are controversial. Some researchers support that it results from pressure on the brachial plexus whereas others advocate that it comprises a neurovascular disorder as both the brachial plexus and subclavian artery are being compressed. This category has also been reported in the literature as common, non-specific, assumed and symptomatic TOS (4, 18, 19).

VTOS is considered a rare form of the syndrome which occurs due to thrombosis of the subclavian or axillary vein following prolonged compression. It has also been referred to as effort thrombosis and Paget–Schroetter syndrome (22). In the occasion of intermittent positional obstruction of the subclavian vein, in the absence of thrombosis, is also referred as McCleery syndrome (23). The literature supports that VTOS affects predominantly men on their dominant hand because of repetitive use of the upper extremity and it accounts for 5% of all TOS cases. However, more recent publications show that its incidence might actually be higher and is estimated at around 20–30% of TOS (9, 11, 14, 15).

The ATOS is reported as an even rarer condition compared to VTOS that occurs from subclavian or axillary artery compression (4, 17, 18). It appears to affect both genders across all age groups. Arterial compression leads to turbulent flow intravascularly, damage of the intima which predisposes in clot formation, distal emboli, local dilation of the artery and aneurysm formation.

Finally, the traumatic TOS is comprised of rare conditions that result in neurovascular compression because of clavicular fracture (4, 24, 25). If the trauma is severe enough then the injury of neurovascular structures can occur in the acute phase along with the clavicular fracture. Traumatic TOS also involves clinical entities that cause delayed injury and pressure to the neurovascular bundle secondary to clavicular trauma. These can be caused by displaced fragments of the injured clavicle, excessive callus formation after non-union of fracture, the development of a growing haematoma or pseudoaneurysm, or even by medical interventions such as fracture manipulation.

In a recent publication, Gharagozloo et al. suggest that the pathophysiology and classification of TOS should be reconsidered following findings from modern dynamic imaging (26). They advocate that neurogenic and venous TOS symptoms are clinical manifestations of the same underlying pathology, which occurs primarily from compression of the subclavian vein. Furthermore, they propose that any form of compression to the neurovascular bundle from cervical ribs or any other last cervical vertebra abnormality (fibrous bands, bone anomalies, or incompletely formed cervical ribs) should not be considered part of TOS but instead should be addressed as an independent syndrome.

**Figure 4:**
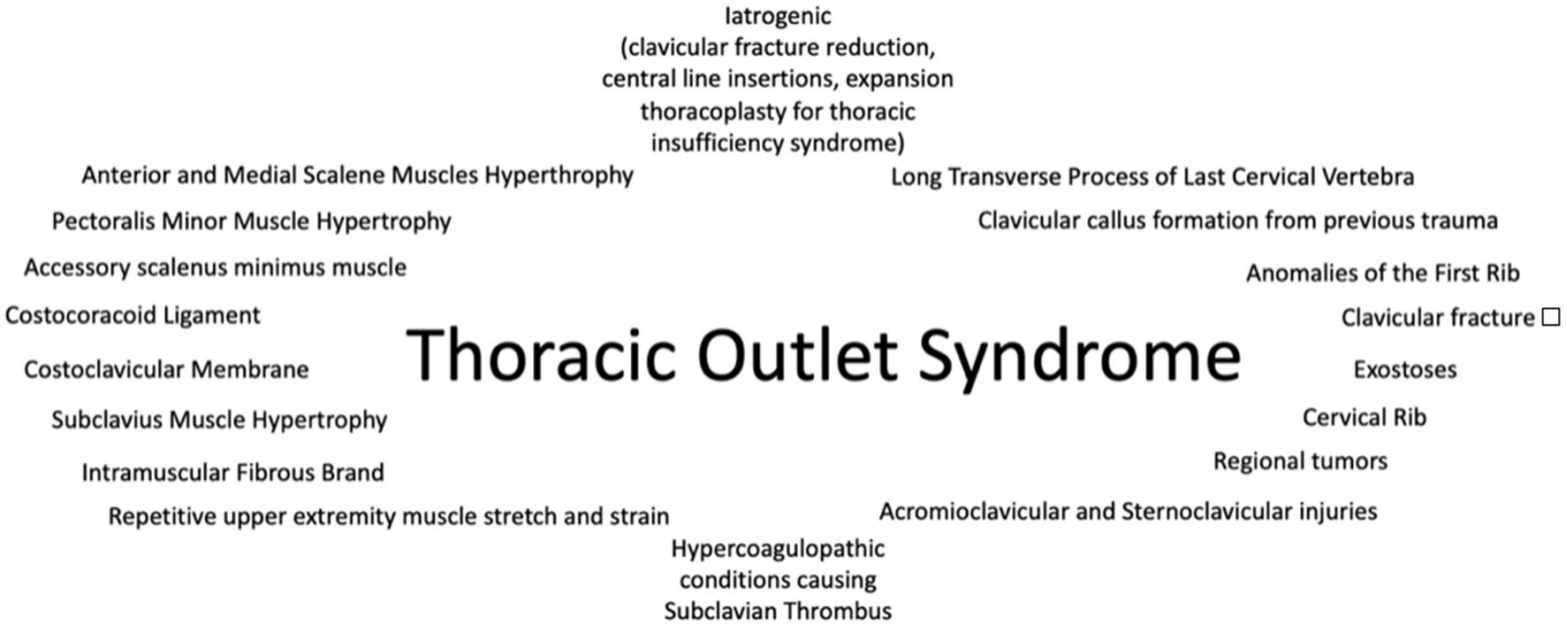
Etiology of thoracic outlet syndrome.

Overall, TOS is comprised of multiple pathological conditions which evoke symptoms in the affected upper extremity (Figure 4). However, inconsistency and disagreements among clinicians and researchers on definitions, diagnostic criteria and pathophysiological mechanisms of the syndrome are posing a challenge for TOS research. The characteristics and different features in TOS classification are summarized in Table 1.

**Table 1:** Classification of TOS.

**Classification**	**Neurogenic**	**Arterial**	**Venous**	**Traumatic**
Subdivision/(alternative terminology)	True (Classic, cervical rib and band syndrome). Disputed (common, non-specific, assumed and symptomatic).		(Paget-Schroetter syndrome, effort thrombosis or McCleery syndrome)	
Prevalence	90–95% or 70–80% of TOS cases. 2–3/100,000 population per year. True NTOS: 1% of the NTOS cases. Disputed NTOS: 99% of the NTOS cases.	1–2% of TOS cases. 0.2–0.7 /100,000 population per year.	5% or 20–30% of TOS cases. 0.5–1/100,000 population per year.	Rare, 1/10^6^ population per year.
Pathophysiology	Compression of brachial plexus cause: 1) Stretch and Angulation of the Lower Brachial plexus. 2) Motor and Sensory Abnormalities of C8 and T1 roots.	Compression of Subclavian and Axillary artery cause: 1) Intima damage. 2) Turbulent flow. 3) Post-stenotic dilatation. 4) Creation of Thrombus. 5) Formation of Aneurysm.	Compression of Subclavian and Axillary vein cause: 1) Damage of the endothelium. 2) Turbulent flow. 3) Restricted vein flow. 4) Creation of Thrombus.	Clavicular trauma that leads to neurovascular bundle compression
Causes (most common)	1) Anterior and/or Middle Scalene Muscle Trauma and Fibrosis (27, 28). 2) Last cervical vertebra anomaly (29). 3) Trauma (whiplash or series of minor cervical injuries). 4) Poor body posture and habitus (long neck with droopy shoulders).	Cervical Rib.	1) Repetitive upper extremity movements (hyperabduction and extension in athletes). 2) Hypercoagulopathic conditions.	Clavicular trauma.
Symptoms	**True NTOS** Motor Abnormalities: Intrinsic hand muscle weakness and wasting (worse in thenar eminence), reduced hand dexterity, impaired fine motor skills. T1 root more affected than C8: Median nerve innervated muscle weakness and wasting. T1 and C8 equally affected: Ulnar nerve innervated muscle weakness and wasting. Sensory abnormalities: Pain, paraesthesia of C8 and T1 dermotome. **Disputed NTOS** Unclear and controversial. predominantly sensory abnormalities. Neck and supraclavicular pain that radiates to the upper limb following lower plexus (C8-T1) or Upper plexus (C5–C6).	**Chronic upper limb ischaemia:** Upper limb pain, claudication, coolness, pallor, decreased CRT, digital ulcers, painless pulsatile subclavian mass and audible bruit. **Acute upper limb ischaemia from distal emboli**	**Acute occlusion:** Diffuse upper extremity swelling, palpable thrombosed axillary veins, upper limb pain, digital cyanosis. **Chronic occlusion:** Dilated veins in the neck, upper chest and shoulder. **McCleery syndrome** Positional and intermittent swelling of the arm.	**Acute trauma:** Pain at the trauma site radiating to the upper extremity, upper extremity edema or loss of peripheral arterial pulse. **Chronic trauma:** Calus palpation on the clavicle at the level of previous injury. Medial cord injury: Sensory medial (1) part of arm, forearm, hand, ring and little finger. Motor (2) Any ulnar and median nerve innervated muscles, except flexor carpi radialis and pronator teres.
Diagnostic tests	1) Sensory and motor nerve conduction studies. 2) Needle EMG. 3) Plain radiography. 4) MRI chest without and with IV contrast (preferable imaging). 5) CT chest (can be considered for post-operative follow-up).	1) Plain radiography. 2) Duplex doppler subclavian artery and vein. 3) Catheter arteriography of the upper extremity. 4) CT angiogram of chest with IV contrast. 5) MRA without and with IV contrast of the chest.	1) Screen for thrombotic disorders. 2) Plain radiography. 3) Duplex doppler subclavian artery and vein. 4) Catheter venography upper extremity. 5) CT chest with IV contrast. 6) MR angiography (disagreement).	1) Plain radiography of chest and clavicle. 2) Sensory and motor nerve conduction studies. 3) CT chest with and without IV contrast. 4) MRI chest without and with IV contrast.

*CRT, Capillary Refill Time; CT, Computer Tomography; EMG, Electromyography; IV, Intravenous; MRA, Magnetic Resonance Angiography; MRI, Magnetic Resonance Imaging; NTOS, Neurogenic Thoracic Outlet Syndrome; TOS, Thoracic Outlet Syndrome*.

### Diagnosis

#### Clinical Findings and Examination

Clinical symptoms of TOS vary according to the underlying etiology (8). It predominantly causes symptoms in the upper extremity which are specific to the components and the level of compression at which the neurovascular bundle is compressed (Table 1). When parts of the brachial plexus are injured or stretched, patients suffer from upper limb heaviness, pain, numbness, intrinsic hand muscle weakness and wasting which are worse on shoulder abduction or palpation above the level of nerve entrapment. In cases of compression to the vein system, patients present with edema and signs of cyanosis in their upper extremity while the superficial veins of the arm, upper chest and neck may appear dilated. By contrast, patients with compressed arterial system suffer from upper limb claudication with numbness and pain on exertion which is relieved at rest. It is important to highlight that a combination of neurological and vascular symptoms is frequently present as different structures of the neurovascular bundle are often compressed. Because of its multivariable underlying pathophysiology, TOS has no pathognomonic signs, tests and symptoms which leads to difficult diagnosis.

The narrow anatomic space of thoracic outlet precipitates the symptoms of the syndrome. Therefore, several maneuvers that compress the thoracic outlet guide clinical diagnosis based on whether they trigger or worsen the symptoms (30). The most commonly used are the elevated arm stress test (EAST), the upper limb tension test (ULTT), the Halsted, Wright, and Adson maneuvers (2, 8, 30). The first two tests aim to provoke symptoms by positioning the upper limb and neck at postures that narrow the thoracic outlet. If symptoms are replicated or exacerbated, then the tests are considered positive. Similarly, the Halsted, Wright, and Adson maneuvers follow the same principle but the clinician palpates the patient's radial artery and assesses if the pulse is decreased in specific postures. However, these tests are not considered gold standard for TOS diagnosis due to published evidence of low specificity as they often have been reported positive in healthy individuals (2, 8).

Due to the complexity and various symptoms linked to TOS, clinicians should appreciate the anatomical characteristics of thoracic outlet when examining and assessing these patients. In that way, symptoms and clinical findings specific to particular structures of the neurovascular bundle justify further investigations in order to diagnose TOS (4, 5, 8). A combination of high level of suspicion, thorough clinical examination and understanding of the syndrome is essential in order to arrange and interpret further clinical investigations (31).

#### Clinical Investigations

In recently published guidelines from the American College of Radiology (ACR), experts reviewed the evidence behind different radiological modalities for TOS diagnosis and post-operative follow-up (31). They reported the indications according to different TOS classifications. Chest Radiography was the only strongly recommended imaging across all TOS classifications. The report also concluded that MRI and MRA of the chest without and with IV contrast is usually appropriate for NTOS and ATOS respectively. On the other hand, different MRI techniques (including MRV, MRA or MRI of the chest +/– IV contrast) were only reported as potentially appropriate for VTOS investigation. CT imaging did not receive a strong recommendation for NTOS due to lack of resolution of neural structures. However, it was regarded as a reasonable approach to assess for adequate decompression in the thoracic outlet post-operatively even in NTOS cases. For cases of vascular involvement, an US duplex Doppler of subclavian artery and vein was strongly recommended while it could also be justified in NTOS investigation and follow-up. Finally, a catheter venography and arteriography of the upper extremity were both considered appropriate imaging for venous and arterial TOS respectively.

For patients presenting with symptoms that indicate compression of the brachial plexus, nerve conduction studies (NCS) and needle electromyography (EMG) provide useful information (4, 5, 32, 33). In cases of true NTOS, EMG and NCS findings that demonstrate a T1 > C8 pattern are even considered pathognomonic when they correlate with the clinical symptoms (20, 29, 32). Comprehensive reports that describe in detail the process of performing sensory NCS, followed by motor NCS and then needle EMG along with MRI have been published and offer a sensible approach for true NTOS diagnosis (5).

Disputed NTOS is much more controversial with regards to its symptoms and diagnosis. The majority of publications support that patients with sensory or motor symptoms suggestive of TOS, should be investigated with NCS and EMG as different clinical entities could be identified (33). On the other hand, others have argued that diagnosis should be solely based on symptoms on the basis of low diagnostic value of those investigations (34, 35). However, there is great discrepancy on what actually constitutes the typical disputed NTOS symptomatology. Some publications report that sensory complaints are usually absent whereas others describe that sensory abnormalities are seen in 90% of the cases (4, 34–36). The latter group, even proceed to categorize them further to patients with upper (C5 or C6 distribution) and lower (C8 or T1 distribution) plexus sensory features (37). Similar disagreement exists on frequency and severity of motor neurological symptoms, with some researchers arguing that muscle weakness is inconsistent with disputed NTOS diagnosis (4, 34–36). Furthermore, (as its name indicates) the diagnosis of disputed NTOS becomes even more challenging due to restrictions to perform accurate and reproducible electrodiagnostic studies that would result in universally accepted findings (4). Even interventional diagnostic procedures that have been developed, such as injecting botulinum toxin, local anesthetics and steroids in the anterior scalene muscle, have not been widely adopted (38, 39). Therefore, given the high variability between symptoms and investigation results, each suspected disputed NTOS case should be evaluated further to exclude other potential etiology.

In an effort to standardize TOS diagnosis and reporting, the Society of Vascular Surgery (SVS) published an executive summary in 2016 (40). This has been adopted by several institutions which have published their experience in managing patients with TOS since then (12, 41). In particular, they suggest that in order to diagnose NTOS at least three out of four criteria are required. These are:

- signs and symptoms occurring at the thoracic outlet,
- peripheral findings (including distal neurologic changes, often worse with provocative maneuvers),
- absence of other pathology predominantly explaining the symptoms (cervical disk disease, shoulder disease, carpal tunnel syndrome, chronic regional pain syndrome, brachial neuritis), and
- positive response to a properly performed scalene muscle test injection.

For VTOS diagnosis, the SVS recommends that a combination of symptoms from medical history and clinical examination (arm swelling, discoloration, heaviness, Table 1) along with radiological findings suggestive of subclavian vein compression is required (40). Furthermore, they specify that if ultrasound or venography findings appear normal at rest, then the investigations should be repeated with the arms abducted >90 degrees. However, they also clarify that if axillosubclavian thrombus is identified on imaging, then VTOS can be diagnosed even in asymptomatic patients (40).

In cases of suspected ATOS, the society suggests that either an injury to subclavian artery or symptoms of upper extremity induced ischemia when the arms are elevated is necessary for the diagnosis. Therefore, it appears that recent consensus for TOS diagnosis suggests that dynamic investigations (duplex ultrasonography, MRV, MRA, Venography, Arteriography) increases their sensitivity (26, 42).

### Treatment and Management

#### Conservative Management

Following TOS diagnosis, the underlying etiology, symptom severity and TOS classification are determined in order to design the appropriate treatment regimen for each patient. This is better performed with collaboration among different medical specialties due to the diverse pathology which surrounds TOS. This team is frequently comprised of neurologists, psychiatrists, radiologists, spine, hand, vascular and thoracic surgeons (12, 43). For patients with NTOS initial treatment is almost always conservative (44). This includes physiotherapy sessions specific for TOS, posture modifications and pain management. If despite conservative management, the patient remains symptomatic then surgical interventions are considered (44, 45).

In cases of VTOS diagnosis, the main goal is to restore the blood flow in the compressed or occluded vein. To achieve this, endovascular therapy is most often indicated with catheter directed thrombolysis, and/or thrombectomy followed by oral anticoagulation. Even though stent insertion could be considered a rational choice for immediate treatment, evidence suggests that it is associated with recurrent thrombosis which is precipitated from the unresolved external pressure to the vessel (46, 47). After completion of a period on anticoagulation, the patients are subsequently referred for First Rib Resection (FRR) in order to release the external pressure to the vein. Currently, there is no universally accepted guidelines on the exact timeframe for surgical referral (47). However, a systematic review, published in 2020, showed that despite the limited data available, decompression of thoracic outlet can be safely performed within 2 weeks from catheter thrombolysis (48). Furthermore, recent publications from experienced centers suggest that an operation should be scheduled at around 2–4 weeks later (12, 49, 50). At that stage, patients should be carefully evaluated for the likelihood to benefit from an operation. Results from a small retrospective study indicate that those who were less likely to improve without an operation were those with recurrent or persisting disease, lengthy duration of symptoms prior to diagnosis, and identifiable structural abnormalities (51). On the contrary, patients with diagnosed hypercoagulopathy and no evidence of venous compression on static or dynamic imaging have less chances to benefit from FRR (4).

The initial treatment plan for the patients who present with acute upper limb ischemia due to distal arterial emboli from thrombus and aneurysm formation in the subclavian artery are similar with VTOS (4). Therefore, the main concern remains to restore the blood flow in the upper limb. That can be achieved with intravenous administration of heparin, endovascular and surgical interventions such as embolectomy. In cases of chronic or critical upper limb ischemia due to ATOS, surgical interventions have been considered the most beneficial treatment options (4, 52). These are typically performed via a supraclavicular approach which offers better access for excision of structures that most often cause arterial compression (FRR, Cervical Rib Resection, Scalenectomy) along with subclavian aneurysm repair or bypass procedure (53–55). However, transaxillary approach has also demonstrated good results for a combination of cervical and first rib resection in ATOS (54).

For the rare occasions of traumatic TOS, the treatment plan is guided from the presence and severity of vascular injury (4). If vascular compromise is detected then surgical management is required whereas in cases of incomplete nerve injury, a more conservative treatment can be considered. The latter includes personalized analgesia, braces and physiotherapy. However, there have also been reports of favorable outcomes with external neurolysis of the brachial plexus in this patient category (34, 56).

#### Surgical Management

Similarly to many other aspects of TOS, there is no universally accepted surgical approach for excising the first rib. These can be categorized to open (10, 57–61), video-assisted (62–69) and robotic-assisted procedures (12, 26, 41–43, 70–77). The traditional procedures for the treatment of FRR have been performed via open surgery using posterior thoracotomy (58, 59), transaxillary (10), supra- or infra-clavicular approaches (61). Each of these techniques offers distinct benefits with regards to exposing specific components in the thoracic outlet. The supraclavicular incision provides direct visualization of the brachial plexus and therefore has been preferred in cases of NTOS. From this approach, the operating surgeon gains access to the thoracic outlet from above and can perform detailed neurolysis and scalenectomy with ease. However, resecting the first rib and especially its anterior third and costoclavicular ligament is notoriously challenging via this approach. The infraclavicular incision provides good exposure of the subclavian vein for patients with VTOS but the posterior part of the first rib cannot be easily excised from under the clavicle. Finally, the transaxillary approach offers increased maneuverability compared to the other two incisions. Since its first publication in 1966 for TOS surgery, it has been considered by many surgeons as the most preferable procedure for a combined cervical and first rib excision (57).

All these techniques were conceptualized and established as standard of practice even before any video-assisted procedures were developed. They are still widely used today and apart from the impressive post-operative outcomes in effectiveness and safety, their supporters highlight some other unique benefits. First, with the open approach, patients do not require chest drain insertion since the chest wall is not opened, the post-operative pain is smaller because the intercostal spaces are not spread, it requires less expensive equipment and the operating surgeons do not require video- or robotic-assisted training. Despite these valid arguments, resecting the first rib on its entirety via an open approach can be technically challenging and requires tissue dissection and mobilization around the brachial plexus, subclavian and axillary vasculature. Injury to these structures can lead to severe complications that could cancel the benefits of the procedure.

For that reason, a thoracoscopic technique was introduced by Ohtsuka et al. (78) in 1999. They proceeded with FRR using video-assisted thoracoscopic surgery (VATS) that allowed them to avoid the neurovascular bundle while they were resecting the first rib on its whole length. Through VATS, the surgeon approaches the first rib from a caudo-cephalad direction and has excellent view of the ribs from within the chest. This offers safer tissue dissection and prevents any injuries to the intercostobrachial cutaneous nerve and components of the neurovascular bundle. The VATS technique has evolved tremendously over the last two decades. Nowadays, high quality instruments such as VATS periosteal elevators, rib nibblers and rib cutters are available. Furthermore, VATS has been adopted more widely around the world and it consists an integral part in cardiothoracic training. Therefore, more surgeons are currently experienced in VATS procedures which increases the chances that the patients will benefit from this approach. However, it is important to note that the available evidence behind VATS FRR is currently based on small retrospective single center studies (64–67, 70, 79).

Another video-assisted FRR technique that has been performed is via transaxillary incision (63, 68, 69, 80, 81). This offers improved visualization of the structures within the thoracic outlet compared to the technique described by Roos in 1966 (10) both for the operating surgeon and the rest of the team which promotes collaboration and teaching. Nevertheless, it still carries some at least theoretical risk of damage to the neurovascular bundle because it requires tissue mobilization and dissection near the brachial plexus and subclavian vessels.

This approach has been thoroughly explored by a team in Ohio, USA. As early as 1985, Martinez et al. published their experience with transaxillary endoscopically-assisted FRR (57). They argued for a safest procedure to decompress the tension on the neurovascular bundle in patients with TOS compared to existing open techniques (57). As the instruments for video-assisted procedures became more sophisticated, the team evolved their practice and kept publishing their experience throughout the years (41, 77). From 2003 and onwards, they used the daVinci Surgical System (Intuitive Surgical, Inc, Sunnyvale, CA) to perform transaxillary FRR. In their most recent publications, they reflect their transition from video-assisted to robotic-assisted FRR in patients with TOS (41, 77). Based on their experience, the authors support the superiority of the robot and explore its potential on specific patient categories.

The first RATS procedure for FRR was described in 2012 by Gharagozloo et al. (72). They initially reported 5 cases of Paget-Schroetter syndrome who had robotic first rib resection (R-FRR) and supported that several attributes of RATS offered significant benefits for FRR. These included the improved maneuverability of the instruments in narrow spaces, offering high-definition magnification and overall better-quality visualization of the operating field (Figure 3). Therefore, Gharagozloo et al. (71) introduced a procedure that combines the benefits of robotic-assisted surgery with the benefits of thoracoscopic surgery for FRR. Approaching the resection of the first rib from within the chest with RATS resulted in the design of a promising operation.

The same group of researchers have published 3 more reviews from their practice with R-FRR for TOS (26, 49, 71). In their most recent report (26), they included 162 patients (83 for VTOS and 79 for NTOS) with a median hospital stay of 3 days (Range: 2–4) for NTOS and median stay of 4 days in patients with Paget-Schroetter syndrome. Furthermore, the Quick DASH Scores were reduced significantly in the immediate post-operative period (5 ± 2.3) and at 6-months (3.5 ± 1.1) compared to the mean pre-operative values of 60.3 ± 2.1 (*p* < 0.0001). In more detail, 71/79 (91%) of the NTOS patients reported that their symptoms were completely resolved in the immediate post-operative period and only 3/79 (3.8%) of them continued to have symptoms at 6 months. With regards to the patients with Paget-Schroetter syndrome, they all had a patent subclavian vein in dynamic MRA even 2 years post-operatively.

These favorable findings led to more wide use of R-FRR for TOS as this is mirrored by the increasing number of relevant publications over the last 5 years (12, 42, 43, 50–52, 62, 73–76). The available evidence suggests that the results from Gharagozloo et al. were reproducible in other departments leading in reduced incidence of morbidity, zero mortality and significant improvement of symptoms in NTOS and rates of subclavian vein patency (Table 2).

**Table 2:** Papers with robotic-assisted first rib resection.

**Publications**	**Year (recruit- ment)**	**Country**	**No of cases**	**TOS classifications**	**Sex (F)**	**Age** **(Mean ±S.D.)**	**Robotic approach**	**Median hospital stay (range)**	**Symptom resolution/SV patency**	**Operation time (Mean** **±S.D.)**	**Complications**
Gharagozloo et al. (71) (Abstract)	2020	USA FL	67	**NTOS: 39** VTOS or PSS: 28	**25 (64%)** 12 (43%)	**34** **±9.5** 24 ± 8.5	Transthoracic	3 (2–4) Days	**Persistent paresthesia at 6months: 2/39 (2.5%)** SV Patent in all patients 24 months post-op with Complete Resolution	87.6 ± 10.8 min	**No neurovascular complications or mortality** 9/28 patients (32%) required endovascular venoplasty after R-FRR.
Gharagozloo et al. (26)	2021	USA FL	162	**NTOS: 79** VTOS or PSS: 83	**50 (63%)** 34 (41%)	**34** **±9.5** 24 ± 8.5	Transthoracic	**3** **(2**–**4)** **Days** 4 Days	**Persistent Pain at 6months: 2/79 (2.5%)** SV Patent in all patients 24 months post-op with Complete Resolution of Symptoms	**87.6** **±10.8 min** 127.6 ± 20.8 min	**No neurovascular complications or mortality** 27/83 patients (31%) required endovascular venoplasty after R-FRR
Gharagozloo et al. (49)	2018 (2010–2015)	USA FL	83	Only PSS	34 (41%)	24 ± 8.5	Transthoracic	4 (2–4) Days	SV Patency at 2 weeks: 57/83 (69%) The other 27 patients showed a patent SV at 3 months following balloon angioplasty +/– stent.	127 ± 20.8 min	No neurovascular complications or mortality
Gharagozloo et al. (72)	2012 (Over 8 months)	USA FL	5	Only PSS	1 (25%)	34.6 ± 10	Transthoracic	3 (2–7)	Complete resolution of symptoms at median 12month F.U.	195 ± 24.6 min	No neurovascular complications. No mortality.
Burt et al. (12)	2021 (2015–2020)	USA TX	66	**NTOS: 69%** VTOS: 31%	47 (71%)	36.0 ± 12.8	Transthoracic	47.8 ± S.D.: 22.0 h	N/R	140.0 ± 57.0 min	Phrenic nerve injury 1 (1%) Sensory Brachial plexus palsy 1 (1%)
Palivela et al. (52)	2021 (2015–2020)	USA TX	90	**NTOS only**	**78 (87%)**	**38** **IQR:** **23**	**Transthoracic**	N/R	**Complete Resolution of Symptoms at 15 weeks**	N/R	N/R
Burt et al. (73)	2020	USA TX	1	**NTOS only**	**1**	**39**	**Transthoracic**	N/R	N/R	N/R	N/R
Kocher et al. (50)	2018 (Jan 2015–Oct 2017)	Switzerland	7	**NTOS: 4** VTOS: 3	4 (57%)	31.5 Range: 29–73	Transthoracic	2 (2–4)	Complete Resolution of Symptoms at 3 months	108 min Range: 80–150	No neurovascular complications. No mortality.
Zehnder et al. (75)	2021 (Jan 2015–Nov 2020)	Switzerland	38	ATOS: 3 VTOS: 20 **NTOS: 4** **Non Specific: 11**	-	-	Transthoracic	2 (1–7)	Complete or Subtotal Resolution of symptoms	133 min Range: 71–120	No neurovascular complications. No mortality.
Zehnder et al. (43)	2021 (Jan 2015–Jul 2021)	Switzerland	23	ATOS: 5 VTOS: 19	16 (70%)	32.5 Range:15–73	Transthoracic	2 (1–4)	Complete (18, 78%) or partial relief (6, 26%) of symptoms	117 min Range: 71–219	No neurovascular complications. No mortality.
Yogeswaran et al. (42)	2020	Belgium	1	VTOS only	1	28	Transthoracic	3 days	Resolution of all symptoms	N/R	No neurovascular complications. No mortality.
Wybaillie et al. (62)	2018	Belgium	1	**NTOS only**	**1**	**35**	**Transthoracic**	**48 h**	**Resolution of all symptoms at 6 weeks**	**105 min**	**No neurovascular complication. No mortality**.
Pupovac et al. (76)	2020	USA NY	17	**NTOS: 8** VTOS: 9	9 (53%)	45 ± 11	Transthoracic	1.8 ± S.D.: 1.9 days	Resolution of symptoms in all patients SV patent in all patients	113.2 ± 55.3 min	No neurovascular complications. No mortality.
Martinez et al. (77)	2005	USA OH	105	**NTOS: 31** N-A-TOS: 58 N-V-TOS: 8 VTOS: 6 Combined: 2	73 (70%)	37.5	Transaxillary	2.8 days	Complete or partial ablation of symptoms occurred in 85%	N/R	Temporary long thoracic nerve dysfunction: 2 (1.9%)
Martinez et al. (41)	2021	USA OH	306 patients 412op	**NTOS: 363** ATOS: 7 VTOS: 45	299op (79%)	37 (13–78)	Transaxillary	N/R	Symptoms Improved	N/R	Neurologic (temporary) Axillary nerve neuropraxia: 2 (0.5%) Long thoracic neuropraxia: 2 (0.5%) Phrenic nerve neuropraxia: 1 (0.2%)
Hoexum et al. (51)	2021	Netherlands	15	Only VTOS	7 (47%)	32.9 (20–54)	Transthoracic (3 patients had conversion to Transaxillary)	3.5 (2–9) Days	Symptoms Improved 91% SV patency at 15.5 months	147.9 min Range: 88–320	No neurovascular complications. No mortality.

*Results from solely Venous Thoracic Outlet Syndrome reported in red, results from solely Neurogenic Thoracic Outlet Syndrome reported in **black**, solely Arterial Thoracic Outlet Syndrome reported in green. ATOS, Arterial Thoracic Outlet Syndrome; F, Female; FL, Florida; F.U., Follow-Up; IQR, Interquartile Range; N-A-TOS, Neurogenic combined with Arterial Thoracic Outlet Syndrome; N/R, Not Reported; NTOS, Neurogenic Thoracic Outlet Syndrome; N-V-TOS, Neurogenic combined with Venous Thoracic Outlet Syndrome; NY, New York; OH, Ohio; Op, Operations; PSS, Paget-Schroetter syndrome; R-FRR, Robotic First Rib Resection; S.D., Standard Deviation; SV, Subclavian Vein; TOS, Thoracic Outlet Syndrome; TX, Texas; USA, United States of America; VTOS, Venous Thoracic Outlet Syndrome*.

The only available study that compared R-FRR with other approaches was a recent study by Burt et al. (12). The researchers compared R-FRR which was performed in 66 patients with FRR via supraclavicular incision in 50 patients. From these patients, 69% had NTOS and 31% VTOS from the R-FRR group while 78% of the patients had NTOS and 22% VTOS from the supraclavicular FRR group. The results showed a statistically significant difference on incidence of brachial plexus palsy between the 2 groups. From the supraclavicular group, 18% of the patients experienced post-operative nerve palsy while that percentage was only 1% in the R-FRR (*p* = 0.002). However, all the brachial plexus palsies that occurred were only temporary. Length of hospital stay and time of operation were not significantly different but the patients in the open surgery group had higher average Visual Analog Scale (VAS) pain score and required greater amount of opioid analgesia during their admission. In a subsequent analysis from the same team, the VAS scores at 2 weeks post-operatively, had a significantly steeper downward trend after R-FRR compared to the patients who were approached with supraclavicular incision (*p* = 0.023). Furthermore, the patients who were operated via RATS, required less frequently opioid analgesia 2 weeks after their operation compared to their counterparts from the supraclavicular group (*p* = 0.002). Overall, the authors acknowledged that following their experience in FRR with both approaches, R-FRR offers exceptional exposure of the first rib without the need to manipulate the brachial plexus or subclavian vessels.

A smaller study published from the Netherlands reached to similar conclusions (51). They provided peri- and post-operative details from their experience with 15 R-FRR in patients with VTOS but also published their results from transaxillary and supra/infra- clavicular approaches. R-FFR had a higher operation time compared to the transaxillary approach but not from the supra/infra- clavicular approach, with 147.9 (88–320) min, 78.5 (44, 80) min and 205.7 (155–309) min respectively. Furthermore, only the supra/infra- clavicular approach appeared to be associated with more complications compared to the other two techniques.

Our cohort analysis appears to show that R-FRR is a safe procedure leading to good clinical outcomes with an acceptable complication rate.

Obviously, certain limitations are present. The most relevant factor that could influence the outcomes of the analysis is the small number of the patients included. Moreover, this analysis is essentially based on a retrospective review of data initially collected for clinical and quality improvement purposes.

In the view of above, R-FRR appears a promising, effective and safe approach for selected patients who suffer from TOS. Even though the available publications for R-FRR have increased over the last 5 years, the level of evidence that they provide remains low. This occurs mainly because that evidence is derived from retrospective analyses of single center experiences. Although this information is useful and contributes to our understanding of the role of surgical interventions in TOS, it does not lead to a definite outcome on which surgical approach should be more widely adopted for FRR in TOS. As most of the relevant publications frequently report modifications in the procedure of R-FRR, low quality comparison arms (not predefined selection criteria for each operation, using historical groups for comparison from past center experience) and great variation among the reported outcomes, we should seek to design higher quality studies in TOS research. To achieve this, we believe that close collaboration is required, especially among high volume and experienced centers in TOS management.

## Conclusions

Thoracic Outlet Syndrome is a complex and controversial clinical entity in almost all its aspects. Surgical intervention is considered as the standard of treatment for patients with persisting or worsening symptoms despite conservative management. Robotic Assisted Thoracoscopic First Rib Resection is a newly developed approach that offers excellent exposure and access to the first rib. The high quality of magnification and incredible maneuverability of the instruments along with the benefits from a thoracoscopic procedure, result in this unique surgical approach for TOS that has been so far proven to be safe and effective. More collaboration is required among experienced centers in TOS management in order to advance research projects on the field that would result in more unambiguous outcomes.

## Author Contributions

FM contributed to conception and design of the study and performed the statistical analysis. FM and AG organized the database. AG wrote the first draft of the manuscript. All authors wrote sections of the manuscript. All authors contributed to manuscript revision, read, and approved the submitted version.

## Conflict of Interest

The authors declare that the research was conducted in the absence of any commercial or financial relationships that could be construed as a potential conflict of interest.

## Publisher's Note

All claims expressed in this article are solely those of the authors and do not necessarily represent those of their affiliated organizations, or those of the publisher, the editors and the reviewers. Any product that may be evaluated in this article, or claim that may be made by its manufacturer, is not guaranteed or endorsed by the publisher.
